# Usefulness of gastric aspirates for diagnosing nontuberculous mycobacteriosis

**DOI:** 10.1038/s41598-023-34948-5

**Published:** 2023-05-15

**Authors:** Masafumi Shimoda, Kozo Morimoto, Takashi Yoshiyama, Yoshiaki Tanaka, Koji Furuuchi, Keiji Fujiwara, Masashi Ito, Masashi Nishimura, Kozo Yoshimori, Ken Ohta

**Affiliations:** 1 Respiratory Disease Center, Fukujuji Hospital, Japan Anti-Tuberculosis Association (JATA), 3–1–24 Mastuyama, Kiyose City, Tokyo 204–8522 Japan; 2 Division of Clinical Research, Fukujuji Hospital, Japan Anti-Tuberculosis Association, Kiyose City, Tokyo Japan; 3 Department of Clinical Mycobacteriosis, Nagasaki University Graduate School of Biomedical Sciences, Nagasaki, Japan

**Keywords:** Microbiology, Clinical microbiology, Infectious-disease diagnostics

## Abstract

Distinguishing between nontuberculous mycobacterial pulmonary disease (NTM-PD) and pulmonary tuberculosis (TB) is difficult. We aimed to evaluate the usefulness of gastric aspirate examination for NTM-PD diagnosis and for differentiating NTM-PD from other diseases, including pulmonary TB. We retrospectively collected data for 491 patients with negative sputum smears or a lack of sputum production at Fukujuji Hospital. We compared 31 patients with NTM-PD to 218 patients with other diseases (excluding 203 with pulmonary TB). Additionally, we compared 81 patients with NTM cultured from at least one sputum or bronchoscopy sample to the other 410 patients. Gastric aspirate examination for NTM-PD diagnosis showed 74.2% sensitivity and 99.0% specificity for culture positivity. There was no significant difference between the nodular bronchiectatic disease and cavitary disease types for culture positivity (*p* = 0.515). The significance of NTM isolation from gastric aspirate showed 64.2% sensitivity and 99.8% specificity for culture positivity. Gastric aspirate examination revealed NTM in one TB patient, allowing TB to be ruled out in 98.1% of patients with NTM cultured from gastric aspirates. Gastric aspirate examination is helpful for early-stage NTM diagnosis and ruling out pulmonary TB. This could lead to more accurate and timely treatment.

## Introduction

Nontuberculous mycobacterial pulmonary disease (NTM-PD) is an infection due to *Mycobacterium* species other than *Mycobacterium tuberculosis* and *Mycobacterium leprae* and is increasingly prevalent worldwide^1^, not only in countries with low tuberculosis (TB) incidence, such as those in North America, Europe, and Australia but also in countries with a medium TB incidence, such as China and Korea^2,3^.

In the epidemiological situation, it is essential to distinguish between NTM-PD and pulmonary TB at an early disease stage due to their similar clinical manifestations^4^. Generally, gastric aspirates are performed in patients who are smear-negative or lack sputum production^5^. If gastric aspirates are helpful for not only TB but also NTM, it could lead to an easy diagnosis of both diseases. Regarding the usefulness of gastric aspirates for NTM-PD diagnosis, a single study reported that its sensitivity and specificity are 63.9–82.4% and 95.8–99.6%, respectively^6^. However, these results were shown by analysing patients with NTM-PD diagnosed by sputum examination and/or bronchoscopy. The interpretation is also uncertain when NTM are cultured from gastric aspirates of patients with suspected mycobacterial infection. Furthermore, the significance of occasional NTM isolation from gastric aspirates and whether TB can be excluded have yet to be clarified.

Accordingly, this study investigated the usefulness of gastric aspirates for diagnosing NTM and differences in the diagnostic accuracy by clinical phenotypes of NTM-PD in smear-negative patients or those without sputum production. In patients with NTM-PD, we investigated the association of gastric aspirate cultures with radiological phenotypes. In addition, we investigated the significance of NTM isolation for ruling out TB by comparing patients identified as having NTM isolation and those with other diseases, including pulmonary TB.

## Methods

### Study design and setting

**Figure 1:**
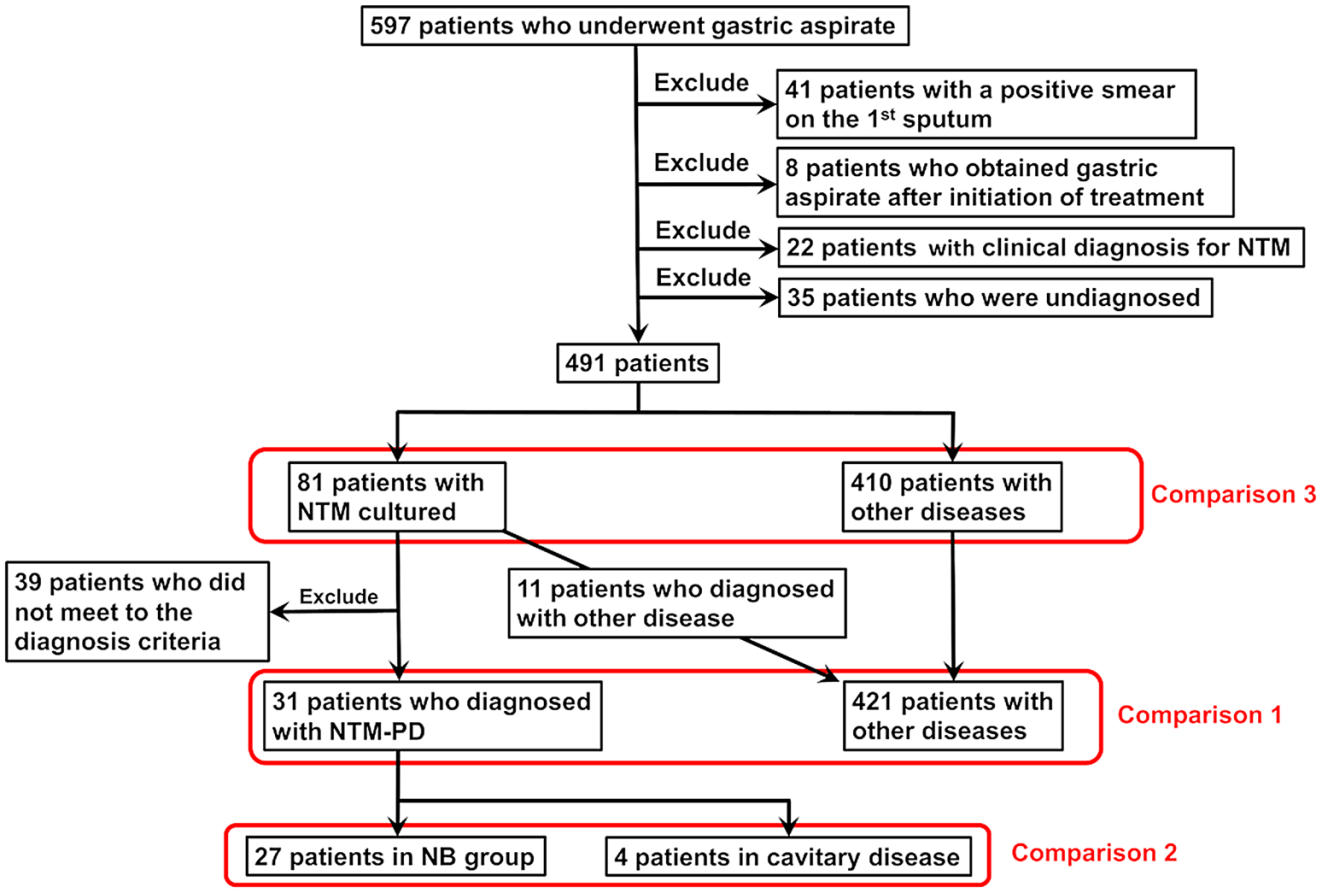
Flowchart of the study. *NTM* Nontuberculous mycobacteria, *NB* Nodular bronchiectatic type, *FC* Fibrocavitary type.

The data of 597 patients who underwent gastric aspirate because they were suspected of having mycobacterial infection at Fukujuji Hospital, Japan Anti-Tuberculosis Association, from January 2016 to March 2021 were collected retrospectively. The flowchart of the study is shown in Fig. 1. Forty-one patients with a positive smear on the 1st sputum examination, eight patients who underwent gastric aspirate examination after receiving anti-TB drugs, 22 patients with a clinical diagnosis of NTM regardless of sputum and gastric aspirate culture negativity, and 35 patients who were undiagnosed and lost to follow-up without a definitive diagnosis were excluded. In total, the data of 491 patients were reviewed. The baseline characteristics of the patients are shown in Table 1. Gastric aspiration was performed once in all patients. There were 81 patients with NTM cultured from at least one sputum or bronchoscopy sample (the NTM culture positivity group) and 410 patients with other diseases (the other diseases group). Patients in the NTM culture positivity group included 31 patients with NTM-PD according to the American Thoracic Society, European Respiratory Society, European Society of Clinical Microbiology and Infectious Diseases, and Infectious Diseases Society of America diagnostic criteria, and the other 50 patients did not meet the criteria through follow-up investigations^7^. The other disease group included 203 patients with pulmonary TB, 58 with pneumonia, 36 with malignant diseases, 30 with radiological findings compatible with cured TB, 27 with inflammatory lesions, 15 with diffuse interstitial lung disease, 12 with bronchiectasis, 6 with mycosis, and 23 with other lung diseases.

**Table 1:** Baseline characteristics of the patients.

Subject	n = 491
Age, median (range), years	67 (18–97)
Sex (Male/Female)	288/202
Diagnosis, n (%)	
NTM-PD	31 (6.3)
NTM (not meet the diagnostic criteria)	50 (10.2)
Pulmonary TB	203 (41.3)
Pneumonia	58 (11.8)
Malignant diseases	36 (7.3)
Radiological findings compatible with cured TB	30 (6.1)
Inflammatory lesions	27 (5.5)
Diffuse interstitial lung disease	15 (3.1)
Bronchiectasis	12 (2.4)
Mycosis	6 (1.2)
Other lung diseases	23 (4.7)

*NTM-PD* Nontuberculous mycobacterial pulmonary disease, *TB* Tuberculosis.

### Subject for comparison

To investigate whether gastric aspirate helps to diagnose NTM-PD, 39 patients who did not meet the NTM-PD diagnostic criteria^7^ were also excluded, as were 11 of 81 patients included in the NTM culture positivity group; therefore, 31 patients with NTM-PD and 421 patients in the other diseases group were compared (Comparison 1). Next, the NTM-PD patients were classified into nodular bronchiectatic (NB) type and cavitary disease (cavitary NB + FC) based on chest computed tomography (CT)^1^. We targeted NB type and cavitary disease because sputum examination in patients with the cavitary disease type is expected to identify NTM specimens more frequently than that in patients with the NB type (Comparison 2)^8^. Finally, to investigate whether gastric aspirate can distinguish patients with gastric aspirate-isolated NTM species from patients with other diseases, including TB, eighty-one patients in the NTM culture positivity group were compared to 410 patients in the other disease group (Comparison 3).

For all patients, the gastric aspirate in the acid-fast bacillus (AFB) smears was subjected to fluorescence staining. Samples from 487 patients were tested using mycobacterial growth indicator tubes (MGITs), and samples from 4 patients were tested using solid medium (egg-based Ogawa) for AFB culture of the gastric aspirate. Samples were incubated at 35 °C in MGITs for 6 weeks and in solid medium for 8 weeks.

### Procedure for collecting gastric aspirate

The collection of gastric aspirate was conducted in a negative pressure room. A sterile, flexible catheter was passed through the patient's nose into the stomach under aseptic precautions, and gastric aspirate was obtained by applying negative pressure with a syringe and transferred to a sterile container. We did not apply irrigation water during the procedure. Prior to the procedure, patients were required to fast for at least 8–10 h.

### Ethical statements

The study was approved by the Institutional Review Board of Fukujuji Hospital. The need for informed consent was waived by the Institutional Review Board of Fukujuji Hospital. The decisions made by this board were based on and in accordance with the Declaration of Helsinki (Study number: 22006).

### Statistical methods

All data were analysed and processed using EZR, version 1.53^9^. The Mann‒Whitney U test and Fisher's exact test were used for group comparisons. The sensitivity, specificity, and odds ratio values were calculated. The McNemar test was used to compare the utility of gastric aspirates and the 1st sputum culture. The level of statistical significance was set at *p* = 0.05 (2-tailed).

## Results

The gastric aspirates of 53 patients identified NTM species, including *M. avium* in 33 patients, *M. intracellulare* in 5 patients, *M. gordonae* in 5 patients, *M. kansasii* in 3 patients, *M. abscessus* in 2 patients, *M. lentiflavum* in 1 patient, and *Mycobacterium* species in 4 patients. Among patients with negative gastric aspirate cultures, *M. avium* was identified among NTM-causing species from sputum or bronchoscopy in 14 patients, *M. intracellulare* in 5 patients, *M. gordonae* in 4 patients, *M. paragordonae* in 2 patients, *M. kansasii* and *M. chelonae* in 1 patient, and *Mycobacterium* species in 2 patients. Only one patient showed discordant NTM species between the gastric aspirate (*M. gordonae*) and sputum (*M. avium*).

In Comparison 1, the median age was 71 years (range 20–90) in patients with NTM-PD and 66 years (range 18–97) in those with the other diseases (*p* = 0.160). *M. avium* in 25 patients, *M. intracellulare* in 4 patients, and *M. kansasii* and *M. abscessus* in 1 patient were identified as the causative bacteria. A difference in the sex ratio was not found between patients with NTM-PD and those with other diseases (males n = 16 [51.6%] vs. n = 252 [59.9%], *p* = 0.476). The diagnostic accuracy of gastric aspirate for NTM-PD was as follows: 38.7% sensitivity and 88.8% specificity for smear positivity and 74.2% sensitivity and 99.0% specificity for culture positivity. The odds ratios of gastric aspirate smear and culture for the diagnosis of NTM-PD were 5.00 (95% Cl 2.07–11.7) and 276.5 (95% Cl 74.6–1356.5), respectively (Table 2). The sensitivity of the 1st sputum culture for patients with NTM-PD was 64.5%, which was lower than that of gastric aspirates, but there was no significant difference (*p* = 0.606). Among 31 patients with NTM-PD, 1 patient was diagnosed by bronchoscopy, and 5 patients were diagnosed by 4 or more sputum examinations.

**Table 2:** Diagnostic accuracy of gastric aspirate examination for NTM-PD.

		NTM-PD	Other diseases	Sensitivity (%)	Specificity (%)	Odds ratio	*p* value
Smear (n = 249)	( +)	12	47	38.7	88.8	5.00 (2.07–11.7)	< 0.001
	(−)	19	374				
Culture (n = 249)	( +)	23	4	74.2	99.0	276.5 (74.6–1356.5)	< 0.001
	(−)	8	417				

*NTM* Nontuberculous mycobacteria.

Table 3 shows comparisons between the NB type and cavitary disease for gastric aspirates (Comparison 2). Among 31 patients with NTM-PD, there were 27 patients with the NB type and 4 patients with cavitary disease. There was no significant difference between the NB type and cavitary disease type (smear n = 10 [37.0%] vs. n = 2 [50.0%], *p* = 0.630; culture n = 19 [70.4%] vs. n = 4 [100.0%], *p* = 0.550).

**Table 3:** Comparisons between the findings of the NB and FC types in patients with NTM-PD on CT scans for gastric aspirate examination.

		NB	Cavitary disease	*p* value
Smear (n = 33)	( +)	10	2	0.630
	(−)	17	2	
Culture (n = 33)	( +)	19	4	0.550
	(−)	8	0	

*NB* Nodular bronchiectatic type, *FC* Fibrocavitary type, *CT* Computed tomography.

In Comparison 3, the significance of gastric aspirate for NTM cultured from at least one sputum or bronchoscopy sample was as follows: 32.1% sensitivity and 88.5% specificity for smear positivity and 64.2% sensitivity and 99.8% specificity for culture positivity (Table 4). Gastric aspirate and sputum examination each identified *M. avium* in one patient with pulmonary TB. Therefore, pulmonary TB could be ruled out in 98.1% of patients who had NTM identified from gastric aspirates. Thirty-one of the 81 (38.2%) patients met the diagnostic criteria for NTM-PD through repeated sputum examination or bronchoscopy. Among 23 patients who could not identify NTM-causing species by cultures from three repeated sputum samples, 78.3% (18 patients) had positive cultures from gastric aspirate, and 33.3% (6 of 18 patients) fulfilled the diagnostic criteria for NTM-PD through follow-up investigations.

**Table 4:** The significance of gastric aspirate for NTM cultured from at least one sputum or bronchoscopy culture (NTM culture positivity group).

		NTM cultured positivity group	Other diseases group	Sensitivity (%)	Specificity (%)	*p* value
Smear (n = 491)	( +)	26	47	32.1	88.5	< 0.001
	(−)	55	363			
Culture (n = 491)	( +)	52	1	64.2	99.8	< 0.001
	(−)	29	409			

*NTM* Nontuberculous mycobacteria.

## Discussion

This study first demonstrates the usefulness of gastric aspirates for NTM-PD diagnosis at an early stage among cases with first smear negativity or those without sputum production. By comparing CT findings, gastric aspiration was useful regardless of the presence or absence of cavity lesions. In addition, it also indicated that NTM isolation from gastric aspirate can distinguish patients from patients with other diseases, including pulmonary TB.

Gastric aspirate can be useful for diagnosing NTM-PD because of its high sensitivity, specificity, and odds ratio. Generally, gastric aspirates are not used for diagnosing NTM-PD because the diagnostic criteria do not include gastric aspirate evaluations^7^. However, in our study, gastric aspirate in NTM-PD was identified as concordant with sputum examination except for one patient who showed different NTM species between the gastric aspirate and sputum, which is consistent with a previous report^10^. Furthermore, NTM-causing organisms were cultured from gastric aspirates of 78.3% (18 of 23 patients) for whom three repeated sputum samples were negative, and 33.3% (6 patients) of those cases fulfilled the diagnostic criteria. Accordingly, the present study indicated that gastric aspirate could be one of the diagnostic tools for NTM-PD for whom diagnosis is difficult based on sputum assessment.

In contrast, when patients swallow contaminated water, their gastric aspirate results can even be smear-positive for AFB regardless of their sputum results^10^; hence, NTM can be isolated due to environmental contamination^7^. Therefore, gastric aspirate for diagnosing NTM should be used in combination with sputum examination to confirm the concordance. Additionally, patients with the NB type generally have a lower bacterial load than those with the cavitary disease type^1^. However, our data showed no significant difference in the NTM positivity rate between the NB type and cavitary type; therefore, it seems that gastric aspirate might be helpful for diagnosing mild NB-type.

The present study also demonstrated the significance of occasional isolation of NTM from gastric aspirates. The study investigated patients who were suspected of having mycobacterial infection but whose first sputum smear was negative or who did not produce sputum, making it difficult to diagnose whether they had pulmonary TB. Our results showed that false-positive gastric aspirates of patients with pulmonary TB were very low (4.9%), similar to sputum examination. Therefore, we believe that gastric aspirate could be used instead of sputum in patients who are suspected of having mycobacterial infection but who cannot provide a sputum sample for examination. If gastric aspirate identifies NTM-causing species, pulmonary TB can be ruled out with high probability, and those patients can be monitored without more invasive examinations such as bronchoscopy or empiric anti-tuberculous treatment. Furthermore, in the present study, six of the 29 patients (21%) who underwent three repeated sputum examinations tested negative, but those for whom gastric aspirate showed a positive result were ultimately diagnosed with NTM-PD by bronchoscopy or by 4 or more sputum examinations.

This investigation had several limitations. The study was conducted retrospectively at a single centre. Gastric aspirates were collected from patients suspected of having a mycobacterial infection. However, the selection of patients who underwent gastric aspirates was made by the clinician, even though it was based on patients with difficulty in obtaining sputum, resulting in variations in patient backgrounds and timing of sample collection. Second, gastric aspirate collection is usually performed in a negative pressure private room in our hospital; however, the risk of contamination with NTM might be increased if gastric aspirate collection is performed where water pollution is present. Therefore, data may vary depending on local water quality. Last, gastric aspiration was performed once in all patients; thus, repeated positive cultures from gastric aspiration were not confirmed. Validation among clinical settings will be needed. Finally, nucleic acid amplification tests of the *M. avium* complex were not performed.

## Conclusions

Gastric aspirate examination is useful for diagnosing NTM-PD at an early stage regardless of the radiological presentation, and it can also help to rule out pulmonary TB in patients at an early disease stage if gastric aspirate shows NTM positivity.

## Data Availability

The datasets used and/or analysed during the current study are available from the corresponding author on reasonable request.
